# Influence of Unusual Co-substrates on the Biosynthesis of Medium-Chain-Length Polyhydroxyalkanoates Produced in Multistage Chemostat

**DOI:** 10.3389/fbioe.2019.00301

**Published:** 2019-11-05

**Authors:** Nils Hanik, Camila Utsunomia, Shuzo Arai, Ken'ichiro Matsumoto, Manfred Zinn

**Affiliations:** ^1^Institute of Life Technologies, University of Applied Sciences and Arts Western Switzerland (HES-SO Valais-Wallis), Sion, Switzerland; ^2^Graduate School of Chemical Sciences and Engineering, Hokkaido University, Sapporo, Japan; ^3^Division of Applied Chemistry, Faculty of Engineering, Hokkaido University, Sapporo, Japan

**Keywords:** medium-chain-length poly(3-hydroxyalkanoate), functional polymer, aromatic polymer, unsaturated polymer, multistage chemostat, steady-state cultivation, carbon flux, chain transfer

## Abstract

A two-stage chemostat cultivation was used to investigate the biosynthesis of functionalized medium-chain-length polyhydroxyalkanoate (mcl-PHA) in the β-oxidation weakened strain of *Pseudomonas putida* KTQQ20. Chemostats were linked in sequence and allowed separation of biomass production in the first stage from the PHA synthesis in the second stage. Four parallel reactors in the second stage provided identical growth conditions and ensured that the only variable was the ratio of decanoic acid (C10) to an unusual PHA monomer precursor, such as 10-undecenoic acid (C11:1) or phenylvaleric acid (PhVA). Obtained PHA content was in the range of 10 to 25 wt%. When different ratios of C10 and C11:1 were fed to *P. putida*, the produced PHA had a slightly higher molar ratio in favor of C11:1-based 3-hydroxy-10-undecenoate. However, in case of PhVA a significantly lower incorporation of 3-hydroxy-5-phenylvalerate over 3-hydroxydecanoate took place when compared to the ratio of their precursors in the feed medium. A result that is explained by a less efficient uptake of PhVA compared to C10 and a 24% lower yield of polymer from the aromatic fatty acid ($y_{\frac{PHA-M}{PhVA}}$ = 0.25). In addition, PHA isolated from cultivations with PhVA resulted in the number average molecular weight $\bar{M_{n}}$ two times lower than the PHA produced from C10 alone. Detection of products from PhVA metabolism in the culture supernatant showed that uptaken PhVA was not entirely converted into PHA, thus explaining the difference in the yield polymer from substrate. It was concluded that PhVA or its related metabolites increased the chain transfer rate during PHA biosynthesis in *P. putida* KTQQ20, resulting in a reduction of the polymer molecular weight.

## Introduction

Tailor-made production of polyhydroxyalkanoates (PHA) bearing unconventional functional groups in the side chain is of high interest since their presence enables the control of the mechanical and physical properties already during biosynthesis (Hany et al., 2005; Tortajada et al., 2013). In addition, these integrated groups enable a further fine-tuning of the PHA using mild, chemical conditions (Levine et al., 2016). ω-Unsaturated and aromatic substrates are examples of unusual PHA precursors (Hartmann et al., 2004). Most of the functionalized biopolyesters are medium-chain-length PHA (mcl-PHA) consisting of C_6_-C_12_ hydroxyalkanoate monomers which have been biosynthesized mainly by *Pseudomonas oleovorans* and *P. putida* (Prieto et al., 2016). Among them, *P. putida* has been shown to be more efficient in the utilization of aromatic carbon substrates for PHA production (Kim et al., 1999).

The wild-type strain *P. putida* KT2440 and its spontaneous rifampicin resistant mutant *P. putida* KT2442 are some of best and most studied producers of mcl-PHA (Follonier et al., 2011; Poblete-Castro et al., 2012). Biosynthesis of mcl-PHA from fatty acids is linked to the fatty acid β-oxidation cycle. Growth substrates that went through β-oxidation build up a pool of different 3-hydroxyalkanoates that can serve for the synthesis of PHA. They differ in their number of C2 units removed during the β-oxidation process and result typically in copolymeric PHAs (Huisman et al., 1989; Ouyang et al., 2007).

In 2011, Liu et al. developed a fatty acid β-oxidation weakened mutant of *P. putida* KT2442, designated as *P. putida* KTQQ20 (Liu et al., 2011). Six key genes of the β-oxidation pathway (*PP2136, PP2137, PP2214, PP2215, PP2047*, and *PP2048*) as well as the 3-hydroxyacyl-CoA-acyl carrier protein transferase (*PP1408*) have been knocked out. This strain still expresses the natural polymerases PhaC1 and PhaC2. The carbon flux is mainly utilized for PHA accumulation. However, the oxidation of fatty acids used as PHA monomer precursors is strongly reduced, rendering *P. putida* KTQQ20 a very suitable strain to study the relationship between substrate and the resulting monomeric unit composition of PHA in detail. Several authors have demonstrated that monomer composition can be tailored to some extent by choosing a specific substrate composition (Hartmann et al., 2006; Liu et al., 2011; Tripathi et al., 2013) using batch, fed-batch or continuous cultivation.

In particular, the continuous cultivation has been identified as a very suitable method to study PHA biosynthesis in a reproducible way (Atlic et al., 2011) once steady-state conditions are achieved. Hartmann et al. found that in contrast to batch cultivation, the chemostat production of PHA in *P. putida* GPo1 results in a time-independent monomeric unit composition when multiple nutrient limited growth was established (Hartmann et al., 2006). The same authors analyzed also in *P. putida* GPo1 the PHA composition when a binary substrate mixture containing *n*-octanoate (C8) and 10-undecenoate (C11:1) was supplied under simultaneous carbon and nitrogen limited growth conditions. Interestingly, they found a non-linear correlation between the fatty acid substrate composition and the 3-hydroxyalkanoates in PHA. In equimolar substrate mixtures, C8 was mainly used as carbon source for biomass formation, thus an increased 3-hydroxy-10-undecenoate content in PHA could be detected.

In this study, we investigated in a two-stage chemostat the accumulation of mcl-PHA in *P. putida* KTQQ20 from unusual substrates as carbon sources in a co-feed experiment. In contrast to the work of Hartmann et al., this experimental set-up allowed the separation of cell growth from accumulation of mcl-PHA precursors. In the first stage, citrate was used as sole carbon source, which was not leading to PHA formation. In the second stage, the transferred cells accumulated PHA because fatty acids were supplied in ample amounts. The weakened β-oxidation leads to PHA monomers structurally related to the supplied fatty acids (i.e., same carbon number) while the co-feed of citric acid in this stage covered maintenance energy requirements of the cells.

Next to composition, the molecular weight of PHAs was shown to play another important role to the mechanical properties of the polymeric materials (Boesel et al., 2014; Arikawa et al., 2016; Huong et al., 2017). Endogenous substrates can influence the molecular weight of the produced polymers by changing the chain transfer rate in the polymerization process (Tomizawa et al., 2010; Tsuge et al., 2013; Hyakutake et al., 2014).

The aim of this work was to study and better understand the substrate kinetics of unusual fatty acids, which are known to strongly influence PHA composition and eventually material properties. Together with the investigation of the effect of unusual substrates on the average molecular weight of the produced polymers, the work aims at influencing the resulting material properties on a molecular level. While the influence of the carbon substrate on the molecular weight was reported elsewhere (Kim et al., 1999; Ward et al., 2006), we propose here for the first time a mechanistic explanation for the observed results based on the kinetic model of the polymerization (Kawaguchi and Doi, 1992).

## Materials and Methods

All chemicals were purchased from Sigma-Aldrich, Switzerland, and used as received if not stated otherwise.

### Cultivation Conditions

**Figure 1:**
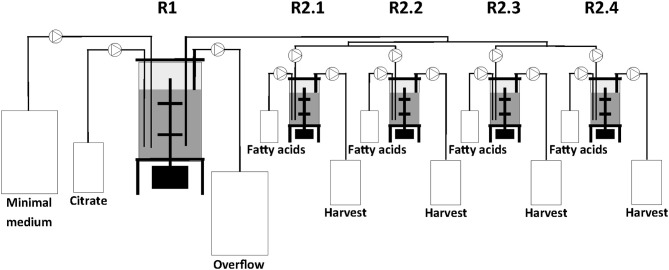
Scheme of the bioreactor set-up showing the combination of serial and parallel reactors.

The two-stage continuous culture system consisted of one 2.5 L (IMCS-2000, newMBR AG, Switzerland) and in sequence a multifermenter with four parallel units of 0.5 L (Sixfors, Infors AG, Switzerland). The reactors had a working volume of 1.25 L and 0.4 L and were designated as the first (R1) and second stage (R2.1, R2.2, R2.3, and R2.4) bioreactors, respectively (Figure 1).

All cultures were performed at 30°C and the pH maintained at 7.0. In R1, cells were grown on the same medium as the preculture. At the end of the batch cultivation in R1, i.e., when the carbon source was fully consumed, the continuous culture system was started. The dilution rates (*D*) of R1 and R2 were 0.1 h^−1^ and 0.2 h^−1^, respectively. The culture volume in R1 was kept constant by continuously transferring the broth to R2 from a tube completely immersed in the culture broth of R1 and by using an overflow tube in R1 both connected to peristaltic pumps. The overflow medium from R1 was collected in a 50 L harvest tank. Cells from R2 were also collected using an overflow tube connected to a continuous running peristaltic pump and the cells were finally kept in a 10 L harvest tank. Usually the steady-states were reached after 5 volume changes (50 and 25 h for *D* = 0.1 h^−1^ and for *D* = 0.2 h^−1^, respectively) and typically PHA production was continued for another 12 h before taking samples. For the different co-substrate feeds, two samples were taken for analyses after steady-state was reached.

### Strain and Media

*Pseudomonas putida* KTQQ20 was kindly provided by Prof. G. Q. Chen (Tsinghua University, China) and used for all experiments (Liu et al., 2011). For inoculum preparation, cells from a frozen stock culture were spread onto LB agar plates and incubated at 30°C for 24 h. The cells were recovered from the plates with a 0.9% NaCl solution and inoculated in 100 mL medium E in 500 mL shake flasks with an initial OD of 0.1. Cells were grown overnight at 30°C and 180 rpm. Medium E contained per liter: 3.5 g NaNH_4_HPO_4_·4H_2_O, 3.7 g KH_2_PO_4_, 7.5 g K_2_HPO_4_, and 2.9 g tri-Na-citrate·2H_2_O. The pH was adjusted to 7.1 with 5 M NaOH. The medium was autoclaved and subsequently supplemented with 1 mL L^−1^ of filter-sterilized 1 M MgSO_4._7H_2_O and 1 mL L^−1^ of mineral trace element stock solution which contained per liter 1 M HCl: 2.78 g FeSO_4_·7H_2_O, 1.47 g CaCl_2_·2H_2_O, 1.98 g MnCl_2_·4H_2_O, 2.38 g CoCl_2_·6H_2_O, 0.17 g CuCl_2_·2H_2_O, 0.29 g ZnSO_4_·7H_2_O.

For continuous cultivation, the following medium was used (per liter): 2.7 g NaNH_4_HPO_4_·4H_2_O, 3.7 g KH_2_PO_4_, and 7.5 g K_2_HPO_4_, supplemented with 1 mL L^−1^ of 1 M MgSO_4._7H_2_O and of mineral trace element stock solution. A total of 40 L of this medium were then filter-sterilized into gamma-sterilized 50 L medium bags (STD FLEXBOY 50 L, Sartorius, Germany). For establishing the dual nutrient (C,N) limited growth regime (DNLGR) with carbon and nitrogen substrates, the amount of NaNH_4_HPO_4_·4H_2_O was reduced to 1.9 g L^−1^ (Egli and Zinn, 2003). The carbon to nitrogen ratio (C_0_/N_0_) of the feed medium was set by the pump flow rates of minimal medium containing ammonium and of a separate feed containing 300 g L^−1^ tri-Na-citrate·2H_2_O as carbon source (Figure 1). Feed solutions containing citrate and different ratios of decanoic acid (C10) and a second fatty acid, such as 5-phenylvaleric acid (PhVA) or 10-undecenoic acid (C11:1) (Tables 1, 2) were pumped directly in R2 using peristaltic pumps (LAMBDA Preciflow, Lambda Instruments, Switzerland). The ratio of citrate to total fatty acids was 2:1 mol mol^−1^.

**Table 1:** Composition of aromatic carbon feed to the second stage bioreactors (R2.1–R2.4).

	**Fatty acid composition in the feed^a^**			
	**100 mol% C10**	**76 mol% C10**	**53 mol% C10**	**0 mol% C10**
Decanoic acid (C10) (mM)	19.1 ± 0.8	14.5 ± 0.6	10.1 ± 0.4	–
5-Phenylvaleric acid (PhVA) (mM)	–	4.6 ± 0.2	9.0 ± 0.4	19.1 ± 0.8

a *The feed of fatty acids was supplemented with a 2-fold molar excess of citric acid to ensure sufficient maintenance energy for the cells, the indicated error is based on an intrinsic error of the analytical method (4%)*.

**Table 2:** Composition of olefinic carbon feed to the second stage bioreactors (R2.1–R2.4).

	**Fatty acid composition in the feed^a^**			
	**100 mol% C10**	**74 mol% C10**	**44 mol% C10**	**0 mol% C10**
Decanoic acid (C10) (mM)	19.1 ± 0.8	14.1 ± 0.6	8.4 ± 0.3	–
10-Undecenoic acid (C11:1) (mM)	–	5.0 ± 0.2	10.7 ± 0.4	19.1 ± 0.8

a *The feed of fatty acids was supplemented with a 2-fold molar excess of citric acid to ensure sufficient maintenance energy for the cells, the indicated error is based on an intrinsic error of the analytical method (4%)*.

### Cell Dry Weight (CDW)

Polypropylene tubes (1.7 mL, Axygen, Corning Inc., Mexico) were dried overnight at 100°C, cooled down in a desiccator over silicagel, and weighed. One mL of culture broth was then added to the tubes and spun down at 21,913 × g at 4°C for 5 min. The culture supernatant was kept at 4°C for further analyses and the cell pellet was washed with 0.9% NaCl solution. The cell suspension was spun down again and the supernatant discarded. The tubes were dried overnight at 100°C and the weight difference was used to calculate the concentration of biomass in the culture.

### Quantification of Substrate Concentrations in the Culture Supernatant

Supernatant resulting from culture centrifugation was filtered through 0.45 μm polyamide filter for all the analyses. Ammonium concentration was measured by using a photometric ammonium test (Spectroquant, Merck, Germany). The detection limit of this method is 0.01 up to 3.00 mg L^−1^ NH_4_-N. If necessary the samples were diluted with demineralized water. Citrate was measured by HPLC-UV equipped with an ion exclusion HPX-87H Aminex column (Bio-Rad, U.S.A.). The analysis was performed in an isocratic mode with 5 mM H_2_SO_4_ as the mobile phase with a flow rate of 0.6 mL min^−1^ for 30 min per sample. C10, C11:1, and PhVa were measured by reversed-phase chromatography equipped with a C18 column (Eclipse XDB-C18, 5 μm, 4.6 × 150 mm, Agilent Technologies) and an UV detector. A gradient of 70% of 0.1% (v v^−1^ formic acid in water to 100% 0.1% (v v^−1^) formic acid in acetonitrile was applied as the mobile phase. The flow rate was 1 mL min^−1^ and the gradient was completed after 15 min.

### PHA Content and Composition

The composition of the polymer and its amount in relation to the biomass was determined as published elsewhere with minor modifications (Furrer et al., 2007). Pyrex vials were weighed to determine the exact transferred biomass (around 30 mg) then 2 mL of methylene chloride containing 2-ethyl-2-hydroxybutyrate (2 g L^−1^) were added as internal standard. Furthermore, 2.5 mL of 1.3 M boron trifluoride-methanol solution were added. The suspension was incubated at 80°C for 20 h. After cooling the samples on ice, 2.5 mL of saturated NaCl solution were added and mixed by vortexing. The water phase was discarded (upper phase), including droplets hanging on the tube wall and including the interface to the methylene chloride phase. The addition of NaCl solution and removal of the water phase was repeated twice. Na_2_SO_4_ and Na_2_CO_3_ were added to dry the methylene chloride phase. The methylene chloride phase was filtered using solvent resistant filters (PTFE, 0.45 μm) and transferred to a GC sample tube. PHA content and monomer composition were subsequently analyzed on a GC (Agilent Technologies, U.S.A.) equipped with a polar fused silica capillary column (DB-WAX: length 30 m; inside diameter 0.25 mm; film thickness 0.25 μm; Agilent Technologies, U.S.A.). Helium was used as carrier gas (3 mL min^−1^) and detection was performed with a flame ionization detector (FID) at 240°C. The temperature was increased from 70 to 240°C at a rate of 10°C min^−1^.

### Determination of PHA Molecular Weight Distribution

mcl-PHA was extracted directly from lyophilized cells. The dried biomass was transferred into pure methylene chloride (40 mg CDW in 10 mL methylene chloride). After stirring the suspension at room temperature for 3 h, the solution was filtered through a filter paper (ashless grade 589/2, white ribbon, pore Size: 4–12 μm, Schleicher&Schüll, Germany). The extracted PHA was subjected to gel permeation chromatography (GPC) for determining the molecular weight distribution. The analysis was performed using a chromatographic system consisting of Waters 171 plus autosampling unit and Waters 515 HPLC pump equipped with an Agilent PLgel MiniMIX-C column and an Agilent 1260 Infinity refractive index detector for the detection of the separated polymers. The system was maintained at 40°C and calibrated with polystyrene standards.

### Calculations

The conversion yield of PHA-monomer from fatty acid ($y_{\frac{PHA-M}{FA}}$) was calculated according to Equation (1) based on the polymer concentration (*c*_*PHA*_), the molecular weight of the fatty acid (*M*_*FA*_) and the one of the monomer (*M*_*monomer*_) which was derived from *M*_*FA*_ by taking oxidation and condensation into account (Equation 2). Equation (1) becomes valid for a high degree of polymerization (DP) considering the fact that the small error introduced by the very first monomer is negligible. The analyzed polymers had a DP between 250 and 600 making this expression for $y_{\frac{PHA-M}{FA}}$ suitable.

The taken up amount of fatty acid (*c*_*FA, uptaken*_) was calculated from the difference of the fatty acid concentration in the feed (*c*_*FA, feed*_) and the residual concentration of the fatty acid found in the supernatant (*c*_*FA, supernatant*_) (Equation 3).

(1) $$\begin{array}{l} y_{\frac{PHA-M}{FA}}=\frac{c_{PHA}\times M_{FA}}{c_{FA,\;uptaken}\times \left(M_{monomer}\right)}\\ \;=\frac{c_{PHA}\times \frac{M_{monomer}}{M_{FA}}}{c_{FA,uptaken}}=\frac{n_{PHA-M}}{n_{FA,uptaken}} \end{array}$$

(2) $$\begin{array}{l} M_{monomer}=M_{FA}+M_{oxygen}-M_{H_{2}O}=M_{FA}-2 \end{array}$$

(3) $$\begin{array}{l} c_{FA,uptaken}=c_{FA,feed}-c_{FA,\;supernatant} \end{array}$$

To calculate the theoretical polymer composition **(**Equation 5**)**, the intracellular fatty acid ratio was calculated from Equation (4) and multiplied with the polymer from fatty acid yield.

(4) $$\begin{array}{r} {\%FA}_{1,uptaken}=100\times \\ \frac{c_{FA1,\;feed}-c_{FA1,supernatant}}{\left(c_{FA1,\;feed}-c_{FA1,supernatant}+c_{FA2,\;feed}-c_{FA2,supernatant}\right)} \end{array}$$

(5) $$\begin{array}{l} Theoretical\;polymer\;composition={\%FA}_{1,\;uptaken}\times y_{\frac{{PHA-M}_{1}}{FA_{1}}} \end{array}$$

The percentile reduction of the number average molecular weight of PHA ($\bar{M_{n}}-reduction$) in the presence of PhVA is described by Equation (6), using the number average molecular weight obtained from GPC analysis for polymers produced either in absence or presence of PhVA ($\bar{M_{n}^{-PhVA}}$ and $\bar{M_{n}^{+PhVA}}$ respectively).

(6) $$\begin{array}{l} \bar{M_{n}}-reduction=100\times \frac{\left(\bar{M_{n}^{-PhVA}}-\bar{M_{n}^{+PhVA}}\right)}{\bar{M_{n}^{-PhVA}}} \end{array}$$

The number of polymer chains per biomass produced (*N*_||*X*||_) was calculated from Equation (7), using the total weight of polymer produced and the corresponding number average molecular weight obtained from GPC analysis. In contrast to the expression derived by Tomizawa et al. (2010), this expression for the chain number takes the different amounts of biomass in the cultivations into account, thus correcting changes in cell densities found in different chemostat cultivations.

(7) $$\begin{array}{l} N_{∥x∥}=\frac{c_{biomass}\times \;\%PHA}{\bar{M_{n}}\times {\left(100-\%PHA\right)\times c}_{biomass}}=\frac{\%PHA}{\bar{M_{n}}\times \left(100-\%PHA\right)} \end{array}$$

The relative number of PHA chains was defined as the number of chains synthesized in the presence of PhVA divided by the number of chains generated in the absence of PhVA (Equation 8).

(8) $$\begin{array}{l} Relative\;number\;of\;PHA\;chains=\;\frac{N_{∥x∥}^{+PhVA}}{N_{∥x∥}^{-PhVA}} \end{array}$$

## Results and Discussion

### Effect of Medium Composition on Polymer Accumulation

The experimental set-up used in this study (Figure 1) allows screening of a multitude of parameters and conditions in a time efficient manner. The parallel set-up of the second stage is provided with identical and stable biomass feeds from the first stage, thus eliminating uncertainties introduced by batch-to-batch changes. Our experiments were designed to study two different growth conditions on the basis of N-limitation in the first (biomass accumulating) stage (R1) while providing comparable series of four different conditions for polymer accumulation in parallel bioreactors of the second stage (R2.1–R2.4) (PHA production stage). The two sets of experiments performed in this study represent one chemostat cultivation under nitrogen-limiting growth conditions during PHA accumulation from the combination of fatty acids C11:1 and C10, and another set with an increased amount of residual nitrogen in the second stage due to higher initial concentration of ammonium when the fatty acids PhVA and C10 were used. As both sets of experiments included the production of poly(3-hydroxydecanoate) (P3HD), the effect of residual nitrogen was reflected by the different polymer contents (wt%) (Tables 3, 4) and different yields of polymer from fatty acid (Tables 5, 6). The increased polymer yield under nitrogen limitation is in good agreement with results reported earlier (Egli, 1991; Zinn and Hany, 2005).

**Table 3:** Polymer accumulation on the fatty acids C10 and C11:1 in R2.

**Polymer composition**	**Dry cell weight (g L^**−1**^)^a^**	**PHA content (wt%)^a^**	**Residual ammonium (mg L^**−1**^)**	**Residual citrate (mg L^**−1**^)^a^**
100% Decanoate	1.1 ± 0.1	15 ± 0.7	nd	14 ± 1
50% Decanoate + 50% 10-undecenoate	1.2 ± 0.1	18 ± 0.4	nd	14 ± 2
75% Decanoate + 25% 10-undecenoate	1.2 ± 0.2	21 ± 0.7	nd	14 ± 1
100% Undecenoate	1.2 ± 0.1	25 ± 0.9	nd	12 ± 6^b^

a *The indicated standard deviation is based on multiple measurements (n = 3)*.

b *n = 2*.

**Table 4:** Polymer accumulation on the fatty acids C10 and PhVA in R2.

**Polymer composition**	**Dry cell weight (g L^**−1**^)^a^**	**PHA content (wt%)^a^**	**Residual ammonium (mg L^**−1**^)^a^**	**Residual citrate (mg L^**−1**^)^a^**
100% Decanoate	1.1 ± 0.1	10 ± 0.1	25 ± 7	12 ± 2
50% Decanoate + 50% PhVA	0.8 ± 0.1	13 ± 0.0	51 ± 2	11 ± 1
75% Decanoate + 25% PhVA	1.2 ± 0.4	12 ± 0.3	41 ± 7	12 ± 2
100% PhVA	0.8 ± 0.1	8 ± 0.3	53 ± 2	12 ± 2

a *The indicated standard deviation is based on multiple measurements (n = 3)*.

**Table 5:** Calculated conversion yield of PHA-monomer from fatty acid for the homopolymer production based on Equation (1).

**Substrates**	**Polymer**	***y*_*PHA*−*M*/*FA*_[mol mol^−1^]**
5-Phenylvalerate	Poly(3-hydroxy-5-phenylvalerate)	0.25
Decanoate	Poly(3-hydroxydecanoate)	0.33

**Table 6:** Calculated conversion yield of PHA-monomer from fatty acid for the homopolymer production based on Equation (1).

**Substrates**	**Polymer**	***y*_*PHA*−*M*/*FA*_[mol mol^−1^]**
10-Undecenoate	Poly(3-hydroxy-10-undecenoate)	0.72
Decanoate	Poly(3-hydroxydecanoate)	0.69

### 10-Undecenoate as Co-substrate

**Figure 2:**
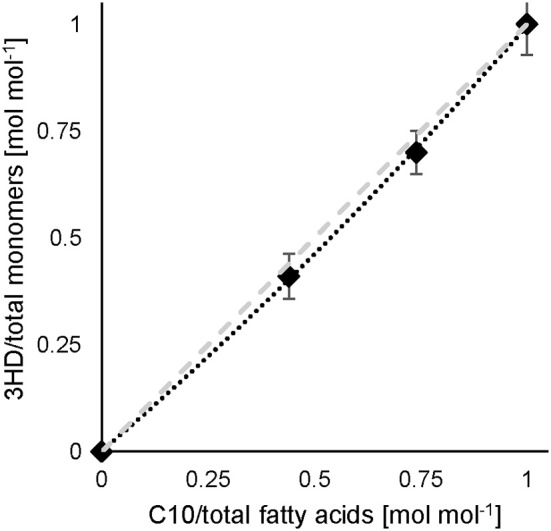
C10 and 10-undecenoate feed and resulting monomer composition. Experimental results (♦) (data see Table S1), dashed line illustrates a linear correlation. Error bars indicate the standard deviation from separate composition analyses by GC (*n* = 3).

In the experiment of co-feeding different mixtures of C11:1 and C10, polymers were obtained representing a slightly higher molar fraction of 3-hydroxy-10-undecenoate monomer (3HUu) than the molar composition of the feed (Figure 2).

This correlation was described earlier for a dual nutrient (C,N) limited growth regime (DNLGR) found for chemostat cultures of *P. putida* GPo1 utilizing different feed compositions of mixtures of C8 and C11:1 (Hartmann et al., 2006). In contrast to the culture on PhVA as co-substrate, no fatty acids were detected in the supernatant during chemostat conditions with C11:1 as co-substrate (data not shown). However, the corresponding $y_{\frac{PHA-M}{FA}}$ for the two fatty acids accounts for the composition of the resulting co-polymers. The higher value for $y_{\frac{PHA-M}{FA}}\;$in homopolymer accumulation from C11:1 indicates that even in this β-oxidation weakened mutant of *P. putida* the carbon flux from C10 as the fatty acid is not restricted to polymer accumulation (Table 6). These results are also in alignment with the calculated $y_{\frac{PHA-M}{FA}}$ and the subsequent determination of the theoretical polymer composition (Table 7).

**Table 7:** Comparison of the theoretical polymer composition calculated from the according conversion yield of PHA-monomer from fatty acid and the experimental results.

**Substrate**	**FA in feed (mol%)**	**FA, uptaken (mol%)**	***y*_*PHA*−*M*/*FA*_[mol mol^−1^]**	**Theoretical polymer composition (mol%)**	**Experimental polymer composition (mol%)^a^**
10-Undecenoate	56	56	0.72	57	58 ± 7
Decanoate	44	44	0.69	43	42 ± 6
10-Undecenoate	26	26	0.72	27	29 ± 2
Decanoate	74	74	0.69	73	71 ± 5

a *The indicated standard deviation is based on multiple measurements (n = 3)*.

These findings fit very well to the observed production of poly(3-hydroxydecanoate-*co*-3-hydroxy-dodecanoate) co-polymers by *P. putida* KTQQ20 on dodecanoate (C12) as the sole source of fatty acids described by Liu et al. (2011), indicating residual β-oxidation activity.

### 5-Phenylvalerate as Co-Substrate

**Figure 3:**
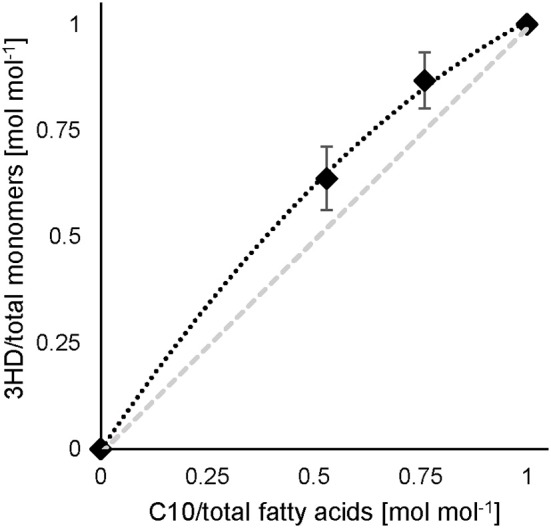
C10 and PhVA feed and resulting monomer composition. Experimental results (♦) (data see Table S2), gray dashed line illustrates a linear correlation. Error bars indicate the standard deviation from separate composition analyses by GC (*n* = 3).

When co-feeding PhVA and C10 in different ratios to the second stage chemostat for PHA accumulation, the resulting polymer composition showed a non-linear correlation between their molar composition of the PHA monomers (Figure 3) and the molar ratio of the substrates fed to the culture. In fact, PhVA is accumulated to a lesser extent than C10. A phenomenon that has been observed as well for simultaneous accumulation of PhVA and *n*-nonanoate (C9) in batch experiments with *Pseudomonas oleovorans* by Kim et al. (1991).

In our experiment, PhVA substrate was detected in the supernatant while C10 was completely consumed. This can be directly correlated to the observed mismatch of fatty acid feed composition and monomer composition in the resulting polymer. However, the extent of differences between these two ratios is still bigger than one would expect considering only the different uptake ratios (Table 8). We therefore performed a flux analysis on the fatty acids in homopolymer production of P3HD and poly(3-hydroxy-5-phenylvalerate) (P3HPhV) to calculate $y_{\frac{PHA-M}{FA}}$. To eliminate the influence of the molecular weight of the monomer as well as the average molecular weight of the copolymer on the yield calculations, we expressed the conversion yield as a molar ratio (Equation 1).

**Table 8:** Comparison of the theoretical polymer composition calculated according to Equation (5) and the experimentally determined composition from GC analysis.

**Substrate**	**c_**FA**_ in feed (mol%)**	**c_**FA**_ uptaken (mol%)**	***y*_*PHA*−*M*/*FA*_[mol mol^−1^]**	**Theoretical polymer composition (mol%)**	**Experimental polymer composition (mol%)^a^**
5-Phenylvalerate	47	43	0.25	36	37 ± 4
Decanoate	53	57	0.33	64	63 ± 8
5-Phenylvalerate	24	23	0.25	18	13 ± 1
Decanoate	76	77	0.33	82	87 ± 7

a *The indicated standard deviation is based on multiple measurements (n = 3)*.

The determined yields of polymer on single fatty acid show a lower value for PhVA than for C10 indicating that in addition to a lower intracellular availability of this fatty acid substrate, its utilization for polymer production is lower than the one from C10 (Table 5). These two major differences in substrate kinetics account for the overrepresentation of 3-hydroxydecanoate (3HD) monomers in the obtained co-polymers. For the mixture with 53 mol% of C10 the theoretical composition fits very well to the experimental results, under consideration of the different uptake as well as the different conversion yield of PHA-monomer from fatty acid (Equation 5).

For the mixture with 76 mol% of C10 the proposed model underestimates the effect observed from the experimental results (Table 8). The differences might be explained by a change in the polymer from fatty acid yield in the presence of a co-substrate.

### Effect of 5-Phenylvalerate on the Polymerization Kinetics

Results of the analyzed $\bar{M_{n}}$ obtained for polymers accumulated in the chemostat cultures were 2-fold reduced for polymers containing 3-hydroxy-5-phenylvalerate (3HPhV) monomers. The molecular weight distribution (PDI) increased with an increasing amount of PhVA in the substrate feed. The obtained $\bar{M_{n}}$ are in good agreement with the results of Kim et al. (1991). Furthermore, in our experiments the amount of polymer accumulated per biomass was independent from the composition of fatty acid substrates (Table 9). While a change in propagation rate would lead to different amounts of polymer per gram of biomass, a higher chain transfer rate of the growing polymer chains for cultures accumulating on PhVA would explain this observation. A mathematical analysis of changes in PHA molecular weight and chain number was performed in analogy to the method proposed by Tomizawa et al. (2010) based on Equations (7) and (8).

**Table 9:** Overview of the mathematical analysis of the molecular weight reduction and the relative chain number of polymer products.

**Substrate composition**	**Dry cell weight** **(g L^**−1**^)^a^**	**PHA content** **(wt%)^a^**	**PHA composition (mol%)**		**Molecular weight**		**$\bar{M_{n}}$ reduction (%)**	**Relative chain number$\frac{N_{\left||x|\right|}^{+PhVA}}{N_{\left||x|\right|}^{-PhVA}}$**
			**3HPhV**	**3HD**	**$\bar{M_{n}}$ (g mol^**−1**^)**	**PDI**		
100% Decanoate	1.1 ± 0.1	10 ± 0.1	0	100	102100	2.0	0	nA
100% 5-Phenylvalerate	0.8 ± 0.1	8 ± 0.3	100	0	50700	4.8	50	1.6
76% Decanoate + 24% 5-phenylvalerate	1.2 ± 0.4	12 ± 0.3	20	80	50000	2.7	51	2.5

a *The indicated standard deviation is based on multiple measurements (n = 3)*.

**Figure 4:**
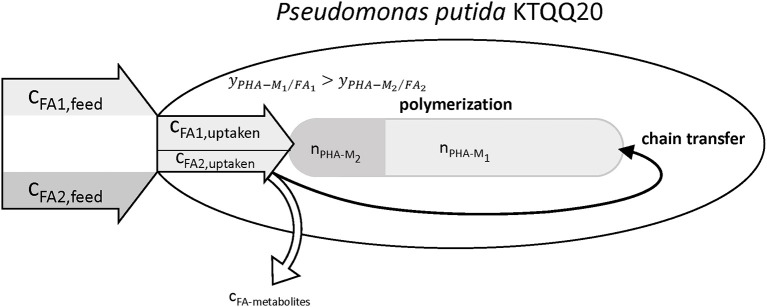
Illustration of the observed effects of PhVA as co-substrate for the accumulation of mcl-polyhydroxyalkanoates. Reduced uptake and lower polymer from fatty acid yields lead to a reduced molar fraction of the corresponding monomer. The metabolites from the non-productive conversion of substrates are suggested to be the reason for the observed increase in chain transfer rate.

The results summarized in Table 9 show values for the relative chain number bigger than one, hence indicating an increased chain transfer rate. In fact, the supernatant of cultures with PhVA contained increased amounts of byproducts linked to PhVA metabolism, which could explain the lower polymer yield from PhVA (Table S3). We consider these metabolites to play an important role on the chain transfer in PHA polymerization (Figure 4) resulting in a polymer with reduced molecular weight. Chain transfer reactions in PHA biosynthesis have been extensively studied for short-chain-length PHA. Addition of exogenous chain transfer agents, mostly alcoholic compounds, in bacterial cultures causes large reduction in PHA molecular weight (Tsuge et al., 2013). Ethanol, on the other hand, has been addressed as a natural chain transfer agent in PHA producing bacteria (Hyakutake et al., 2014).

## Conclusion

Our results show that the presence of PhVA leads to an increased chain transfer rate during polymerization of mcl-PHA by *P. putida* KTQQ20. We have found that the two-stage chemostat is a versatile platform with a high degree of control over important process parameters giving new insights into the mechanism of PHA biosynthesis with reduced molecular weights. Our future work will be dedicated to investigate the role of PhVA in the increased chain transfer rate together with its lower conversion yield by identifying its metabolic fate.

## Data Availability Statement

The raw data supporting the conclusions of this manuscript will be made available by the authors, without undue reservation, to any qualified researcher.

## Author Contributions

NH, CU, and MZ contributed conception and design of the study. NH, CU, and SA organized the database. NH wrote the first draft of the manuscript. NH, CU, and MZ wrote sections of the manuscript. All authors contributed to manuscript revision, read and approved the submitted version.

### Conflict of Interest

The authors declare that the research was conducted in the absence of any commercial or financial relationships that could be construed as a potential conflict of interest.
