# Supplementary material for: Influence of Unusual Co-substrates on the Biosynthesis of Medium-Chain-Length Polyhydroxyalkanoates Produced in Multistage Chemostat
Source: Front Bioeng Biotechnol. 2019 Nov 5;7:301. doi: 10.3389/fbioe.2019.00301 (PMC6848797; doi:10.3389/fbioe.2019.00301)
Supplement: Supplementary file 1 [file Table_1.docx]

Supplementary Material

Table S1. Feed composition and monomer composition expressed as decanoate in mol mol^-1^ in the binary mixture with C11:1.

| Feed composition [mol mol^-1^ C10] | Monomer composition [mol mol^-1^ 3HD] |
| --- | --- |
| 0.00 | 0.00 |
| 0.44 | 0.41 |
| 0.74 | 0.70 |
| 1.00 | 1.00 |

| feed composition  [mol mol^-1^ C10] | monomer composition [mol mol^-1^ 3HD] |
| --- | --- |
| 0 | 0 |
| 53 | 64 |
| 76 | 87 |
| 100 | 100 |

**Table S2.** Feed composition and monomer composition expressed as C10 in mol mol^-1^ in the binary mixture with PhVA.

**Table S3.** Signal intensities from HPLC-peaks indicating a byproduct in the presence of PhVA in the fatty acid feed.

| Fatty acid composition | Retention time [min] | Signal intensity [mAU] |
| --- | --- | --- |
| 100% Decanoate | - | nd |
| 53% Decanoate + 47% 5-Phenylvalerate | 4.8 | 950 |
| 76% Decanoate + 24% 5-phenylvalerate | 4.8 | 618 |
| 100% 5-Phenylvalerate | 4.8 | 1098 |
